# Prognostic impact of preoperatively elevated and postoperatively normalized carcinoembryonic antigen levels following curative resection of stage I‐III rectal cancer

**DOI:** 10.1002/cam4.2758

**Published:** 2019-12-04

**Authors:** Yuya Nakamura, Dai Shida, Taro Tanabe, Yasuyuki Takamizawa, Jun Imaizumi, Yuka Ahiko, Ryohei Sakamoto, Konosuke Moritani, Shunsuke Tsukamoto, Yukihide Kanemitsu

**Affiliations:** ^1^ Department of Colorectal Surgery National Cancer Center Hospital Tokyo Japan

**Keywords:** carcinoembryonic antigen, postoperative CEA, preoperative CEA, prognostic factor, rectal cancer

## Abstract

**Background:**

Preoperative and early postoperative serum carcinoembryonic antigen (CEA) levels are known prognostic factors in rectal cancer. Recently, a large‐scale study on colon cancer revealed that “preoperatively elevated and postoperatively normalized CEA levels” is not an indicator of poor prognosis. However, whether this hold true in rectal cancer patients is unknown. This study aimed to investigate the prognostic significance of preoperatively elevated and postoperatively normalized CEA levels in rectal cancer patients undergoing curative resection.

**Methods:**

Subjects were consecutive stage I‐III rectal cancer patients who underwent curative resection without preoperative treatment at National Cancer Center Hospital between 2000 and 2015. Overall survival (OS) and the hazard function of recurrence or death were analyzed according to the CEA levels, as follows: normal preoperative CEA (normal group), preoperatively elevated but postoperatively normalized CEA (normalized group), and preoperatively and postoperatively elevated CEA (elevated group).

**Results:**

The normalized group (n =235) had worse OS (HR 1.49, 95% CI 1.08‐2.04; *P *= .0142) compared to the normal group (n = 1208), and better OS compared to the elevated group (n = 47) (HR 0.53, 95% CI 0.31‐0.91; *P* = .0208). The elevated group had the highest and earliest peak in hazard function, followed by the normalized group and the normal group, with median times to recurrence of 8.8, 15.5, and 18.5 months, respectively (*P* = .0223).

**Conclusions:**

Prognosis after resection of rectal cancer was worse in patients with preoperatively elevated and postoperatively normalized CEA compared to those with normal preoperative CEA. Patients with elevated preoperative CEA might require intensive follow‐up even if levels normalize after resection, especially in earlier periods, for early detection of recurrence.

## Graphical abstract

Recently, a large‐scale study on colon cancer revealed that “preoperatively elevated and postoperatively normalized CEA levels” is not an indicator of poor prognosis. In this study, we demonstrated that, in rectal cancer, “preoperatively elevated and postoperatively normalized CEA levels” is a definitive indicator of poor prognosis.

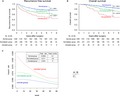

## 1 INTRODUCTION

Guidelines of the National Comprehensive Cancer Network (NCCN), European Society for Medical Oncology (ESMO), and American Society of Clinical Oncology (ASCO) recommend preoperative workup and postoperative measurements of serum carcinoembryonic antigen (CEA) every 3‐6 months for patients with rectal cancer who undergo curative resection.^1^, ^2^, ^3^ Both preoperative and early postoperative CEA levels are known to be prognostic factors after curative resection for rectal cancer, with elevated preoperative CEA^3^, ^4^, ^5^ as well as continuously elevated postoperative CEA being associated with worse survival.^6^, ^7^, ^8^

In a recent study, recurrence‐free survival (RFS) of patients with postoperatively normalized CEA was shown to be similar to that of patients with normal preoperative CEA,^9^ suggesting that perioperative change in CEA, rather than preoperative level of CEA, is important in predicting prognosis in colon cancer. Meanwhile, rectal cancer differs from colon cancer in terms of survival and recurrence patterns,^10^ and thus, changes in CEA levels after curative resection might also differ.^8^ In this regard, the prognostic significance of preoperatively elevated but postoperatively normalized CEA in rectal cancer warrants further investigation.

Preoperative treatment such as chemoradiotherapy and chemotherapy prior to total mesorectal excision is the current standard for locally advanced rectal cancer in many Western countries.^11^ Meanwhile, in Japan, upfront surgery (total mesorectal excision plus lateral lymph node (LN) dissection) without any preoperative therapy is performed as the standard treatment for rectal cancer.^12^ This unique strategy in Japan allows for serial evaluation of perioperative CEA levels in patients with rectal cancer, without any influence of preoperative therapy.

This study aimed to investigate the prognostic impact of preoperative and early postoperative serum CEA in patients with stage I‐III rectal cancer who underwent curative resection, focusing mainly on the prognostic significance of preoperatively elevated and postoperatively normalized CEA following curative resection.

## 2 MATERIALS AND METHODS

### 2.1 Study population and design

The study population comprised patients with stage I‐III rectal adenocarcinoma who underwent curative resection at National Cancer Center Hospital in Japan from January 2000 to December 2015. According to the Japanese classification of colorectal, appendiceal, and anal carcinoma, the rectum is defined as the segment from the height of the inferior border of the second sacral vertebra to the superior border of the puborectal sling.^13^ Patients who underwent preoperative treatment such as chemoradiotherapy or chemotherapy were excluded, given the possible influence on preoperative serum CEA. Patients with elevated preoperative CEA, for whom early postoperative CEA measurements were not available, were also excluded from the analysis.

Preoperative CEA was defined as CEA measured immediately prior to surgery, and early postoperative CEA was defined as CEA measured within 3 months after surgery and before adjuvant chemotherapy. The reference range of CEA was set at ≤5 ng/ml. Patients were divided into the following three groups according to levels of preoperative and early postoperative CEA: normal preoperative CEA (normal group), elevated preoperative and normalized postoperative CEA (normalized group), and elevated preoperative and postoperative CEA (elevated group). RFS and overall survival (OS) were investigated in each group.

The Institutional Review Board (IRB) of the National Cancer Center Hospital approved this retrospective study (IRB code: 2017‐437).

### 2.2 Follow‐up

Postoperative follow‐up consisted of serum CEA and CA19‐9 measurements every 3 months for the first 2 years, then every 6 months for 3 years; computed tomography (CT) every 6 months for 5 years; and colonoscopy in the first and third year, as described previously.^14^ Follow‐up data were documented prospectively until an event occurred, or until the study cutoff date of August 2018.

### 2.3 Statistical analysis

Pearson's Chi‐square test and the Kruskal‐Wallis test were performed for categorical variables and continuous variables, respectively, to examine various factors in each group. Continuous variables are presented as median (interquartile range). RFS was defined as the interval between the date of surgery of primary cancer and the date of recurrence or death from all causes. OS was defined as the interval between the date of surgery of primary cancer and the date of death from all causes. Patients alive at the end of the follow‐up period were censored. The Kaplan‐Meier method was used to estimate RFS and OS. Differences in survival were assessed with the log‐rank test. Multivariable Cox proportional hazards regression models were subsequently fitted to evaluate factors independently associated with recurrence and death. Selection of covariates to be included in the multivariable analysis was performed based on information from previous studies.^5^, ^15^ The hazard function (smoothed hazard estimate) of recurrence or death, which conveys the instantaneous conditional recurrence or death rate at time *t*, was also analyzed 16, 17 and expressed as the number of events per unit of time (months). Propensity score matching analysis was conducted in order to balance the distribution of covariates between the normal preoperative CEA group and elevated preoperative CEA group (which included the normalized and elevated subgroups), as described previously.^18^, ^19^ Multivariable logistic regression was used to generate propensity score‐predicting preoperative CEA status based on confounding covariates, including sex (male vs female), procedure (sphincter‐preserving vs non‐preserving), lymphatic invasion (present vs absent), venous invasion (present vs absent), and TNM stage (I/II vs III). Propensity scores were used for matching, which pair patients with normal preoperative CEA and patients with elevated preoperative CEA according to similarities in their baseline characteristics. Each patient with normal preoperative CEA was matched 1:1 with a patient with elevated preoperative CEA using the closest estimated propensity on the logit scale within a specified range (smaller than 0.05 of estimated logits as the caliper width) in order to reduce differences between the two groups, and outcomes in matched patients were compared and analyzed.

*P *< .05 was considered statistically significant, and 95% confidence intervals (CIs) were calculated. Bonferroni correction for multiple comparisons was performed to compare outcomes. All statistical analyses were performed using the JMP14 software program (SAS Institute Japan Ltd., Tokyo, Japan) or R version 3.5.3 (R Project).

## 3 RESULTS

### 3.1 Study cohort characteristics

**Figure 1:**
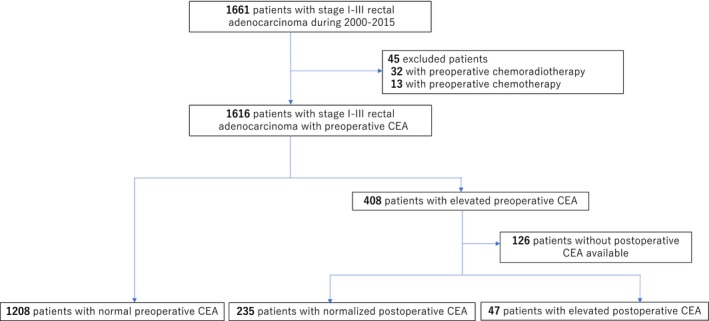
CONSORT diagram for patient selection. After exclusion of patients who underwent preoperative treatment (n = 45) and those with elevated preoperative CEA, those for whom postoperative CEA measurements were not available (n = 126), from the initially recruited patients with stage I‐III rectal cancer (n = 1661), the final number of analyzed patients was 1490

Figure 1 shows the flowchart of patient selection and classification into three groups according to levels of preoperative and postoperative CEA. A total of 1661 stage I‐III rectal cancer patients who underwent curative resection were identified. Of these, 1490 patients were subjected to analysis, excluding 45 who underwent preoperative chemoradiotherapy or chemotherapy, and 126 with elevated preoperative CEA, for whom early postoperative CEA measurements were not available. Data on preoperative CEA were obtained from all patients; 1208 had normal preoperative CEA, 235 had elevated preoperative CEA which subsequently normalized postoperatively, and 47 had elevated preoperative CEA which persisted postoperatively.

Table 1 summarizes patient clinicopathologic features by group. Patients with elevated postoperative CEA (elevated group) were older (*P* = .0016) and had poor tumor differentiation (*P* = .002), and those with elevated preoperative CEA (both the normalized and elevated groups) had more venous invasion (*P* < .0001) and advanced tumor stage (*P* < .0001). The tumor distance from the anal verge was longer in the normal group (*P* = .0197), and sphincter‐preserving surgery was performed more frequently in the normalized group (*P* < .0001).

**Table 1:** Patient characteristics

	All	Normal CEA (N = 1208)	Normalized CEA (N = 235)	Elevated CEA (N = 47)	*P* value
Age (years; median, IQR)	62 (54, 69)	62 (54, 68)	61 (52, 68)	68 (59, 73)	.0016
Operation year					.992
2000‐2007	816 (55)	662 (55)	128 (54)	26 (55)	
2008‐2015	674 (45)	546 (45)	107 (46)	21 (45)	
Sex					.647
Male	1001 (67)	806 (67)	161 (69)	34 (72)	
Female	489 (33)	402 (33)	74 (31)	13 (28)	
Tumor distance from the anal verge, cm					.0197
≤5	670 (45)	523 (43)	125 (53)	22 (53)	
>5	820 (55)	685 (57)	110 (47)	25 (47)	
Clinical stage					<.0001
I	544 (37)	518 (43)	18 (8)	8 (17)	
II	392 (26)	302 (25)	71 (30)	19 (40)	
III	554 (37)	388 (32)	146 (62)	20 (43)	
Operative procedure					<.0001
Sphincter‐preserving	1246 (84)	1040 (86)	168 (71)	38 (81)	
Non‐preserving	244 (16)	168 (14)	67 (29)	9 (19)	
Tumor differentiation					.0015
Well	803 (54)	681 (56)	98 (42)	24 (51)	
Moderate	615 (41)	472 (39)	125 (53)	18 (38)	
Poor	71 (4)	54 (4)	12 (5)	5 (11)	
Not available	1 (0.07)	1 (0.08)	0	0	
Lymphatic invasion					.1204
Yes	457 (30)	352 (29)	87 (37)	18 (38)	
No	1028 (69)	852 (70)	147 (62)	29 (62)	
Not available	5 (0.34)	4 (0.33)	1 (0.43)	0	
Venous invasion					<.0001
Yes	828 (55)	626 (52)	166 (71)	36 (77)	
No	656 (44)	576 (47)	69 (29)	11 (23)	
Not available	6 (0.40)	6 (0.50)	0	0	
No. of retrieved LNs, median (IQR)	28 (19, 41)	26 (18, 39)	38 (26, 52)	30 (21, 43)	<.0001
T category					<.0001
T1	355 (24)	347 (28)	6 (2)	2 (4)	
T2	375 (25)	338 (28)	30 (13)	7 (15)	
T3	690 (46)	480 (40)	176 (75)	34 (72)	
T4	70 (5)	43 (4)	23 (10)	4 (9)	
N category					<.0001
N0	877 (59)	771 (64)	86 (37)	20 (43)	
N1	377 (25)	283 (23)	79 (34)	15 (32)	
N2	236 (16)	154 (13)	70 (30)	12 (25)	
TNM stage (UICC 8th)					<.0001
I	564 (38)	530 (44)	25 (11)	9 (19)	
II	313 (21)	241 (20)	61 (26)	11 (23)	
III	613 (41)	437 (36)	149 (63)	27 (58)	
Circumferential resection margin					.1979
Negative	1438 (97)	1171 (97)	222 (94)	45 (96)	
Positive	52 (3)	37 (3)	13 (6)	2 (4)	
Adjuvant chemotherapy					<.0001
Yes	367 (25)	257 (21)	100 (43)	10 (21)	
No	1123 (75)	951 (79)	135 (57)	37 (79)	
Preoperative CEA, median (IQR)	2.6 (1.6, 4.4)	2.3 (1.5, 3.3)	10.7 (7.1, 23.7)	11.4 (7, 42.3)	<.0001

Note

Data are expressed as n (%) unless otherwise specified.

Abbreviations: CEA, carcinoembryonic antigen; LN, lymph node.

Fourteen patients received postoperative radiation. Of these, seven had positive CRM, three had lateral LN metastases, and four underwent transanal resection. Twelve patients had normal preoperative CEA levels and two had elevated preoperative but normalized postoperative CEA levels.

### 3.2 Long‐term outcomes after curative resection

**Figure 2:**
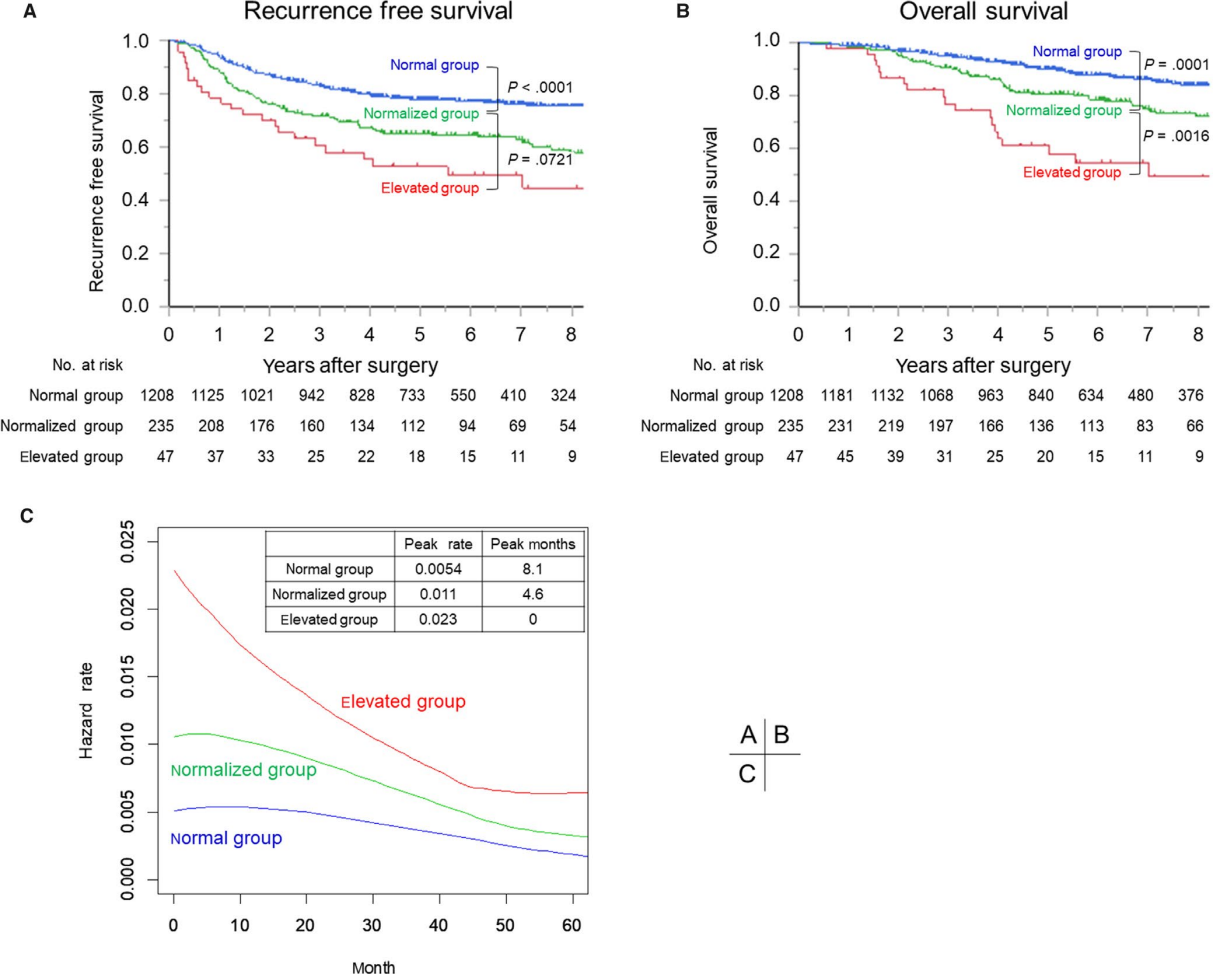
Recurrence‐free survival curves (A) and overall survival curves (B) for the normal group (normal preoperative CEA), normalized group (elevated preoperative and normalized postoperative CEA), and elevated group (elevated preoperative and postoperative CEA). C, Hazard function curves for each group, indicating the instantaneous conditional recurrence or death at time *t*

Figure 2A,B show RFS and OS curves by group (*P* < .0001 and *P* < .0001, respectively). The 5‐year RFS and 5‐year OS rates were 78.0% and 90.7%, respectively, for the normal group (n = 1208), 65.1% and 80.5%, respectively, for the normalized group (n = 235), and 49.6% and 61.1%, respectively, for the elevated group (n = 47).

Bonferroni correction for multiple comparisons revealed a worse RFS in the normalized group compared to the normal group (*P* < .0001), and no significant difference in RFS between the normalized group and elevated group (*P* = .0721). Moreover, the normalized group had a worse OS than the normal group (*P *= .0001), but a better OS than the elevated group (*P* = .0016).

### 3.3 Hazard functions for disease recurrence

Figure 2C shows hazard function curves by group. These curves represent the instantaneous risk for the event at each time‐point, whereas Kaplan‐Meier survival curves represent the cumulative risk. The elevated group had the highest and earliest peak in hazard function (peak rate: 0.023, peak months: 0), followed by the normalized group (peak rate: 0.011, peak months: 4.6) and the normal group (peak rate: 0.0054, peak months: 8.1). The median time to recurrence from operation was shortest for the elevated group at 8.8 months, followed by the normalized group (15.5 months) and the normal group (18.5 months) (Table 3; *P* = .0223).

### 3.4 Stage‐specific long‐term outcomes after curative resection

The 5‐year OS rate was 95.5% (95% CI 93.2‐97.0) for 564 stage I patients, 90.2% (95% CI 86.1‐93.1) for 313 stage II patients, and 79.4% (95% CI 75.9%‐82.6%) for 613 stage III patients (Table 2).

**Table 2:** Univariable and multivariable analyses of overall survival

	No. of patients	5‐year OS	95% CI	*P*	HR	95% CI	*P*
Age, y							
<65	906	90.2	88.0‐92.0	<.0001	Reference		
≥65	584	83.6	80.2‐86.6		1.95	1.50‐2.54	<.0001
Operation year							
2000‐2007	816	85.4	82.8‐87.7	.0006	1.67	1.21‐2.29	.0017
2008‐2015	674	91.0	88.2‐93.1		Reference		
Sex							
male	1001	86.0	83.5‐88.1	.0021	1.57	1.16‐2.13	.0033
female	489	91.3	88.2‐93.6		Reference		
Tumor distance from the anal verge, cm							
≤5	670	85.9	82.9‐88.5	.0284	1.23	0.952‐1.59	.1125
>5	820	89.0	86.5‐91.0		Reference		
Tumor differentiation							
well	803	91.7	89.5‐93.5	<.0001	Reference		
moderate	615	84.7	81.5‐87.5		1.32	1.00‐1.74	.0479
poor	71	66.6	53.5‐77.5		1.71	1.06‐2.77	.0276
Lymphatic invasion							
yes	457	80.7	76.7‐84.2	<.0001	1.59	1.20‐2.09	.0010
no	1028	90.8	88.8‐92.5		Reference		
Venous invasion							
yes	828	84.0	81.2‐86.5	.0001	1.27	0.954‐1.70	.1012
no	656	92.1	89.7‐94.0		Reference		
No. of retrieved LNs							
≥12	1380	87.6	85.6‐89.3	.815	Reference		
<12	110	89.4	81.9‐94.0		1.46	0.904‐2.37	.1215
TNM stage (UICC 8th)							
I	564	95.5	93.2‐97.0	<.0001	Reference		
II	313	90.2	86.1‐93.1		1.78	1.14‐2.80	.0118
III	613	79.4	75.9‐82.6		3.11	2.04‐4.74	<.0001
Circumferential resection margin							
Negative	1438	88.2	86.3‐90.0	.0015	Reference		
Positive	52	73.5	58.8‐84.4		1.79	1.03‐3.12	.0400
Adjuvant chemotherapy							
yes	367	83.0	78.6‐86.7	.0074	0.786	0.565‐1.09	.1529
no	1123	89.2	87.1‐91.0		Reference		
CEA group							
normal	1208	90.7	88.1‐91.7	<.0001	Reference		
normalized	235	80.5	74.5‐85.3		1.45	1.06‐2.00	.0211
elevated	47	61.1	45.4‐74.7		2.64	1.62‐4.29	<.0001

Abbreviations: OS: overall survival, CI: confidence interval, HR: hazard ratio, LN: lymph node, CEA: carcinoembryonic antigen.

Among stage III patients, stage‐specific RFS and OS rates significantly differed by group (RFS: HR 1.52, 95% CI 1.14‐2.03 for normalized vs normal groups (*P* = .0042) and HR 2.38, 95% CI 1.47‐3.87 for elevated vs normal groups (*P* = .0004); OS: HR 1.50, 95% CI 1.03‐2.18 for normalized vs normal groups (*P* = .0350) and HR 3.23, 95% CI 1.85‐5.63 for elevated vs normal groups (*P *< .0001)) (Figure S1). On the other hand, among stage I/II patients, both stage‐specific RFS and OS rates showed no significant differences by group (Figure S1).

### 3.5 Factors affecting prognosis after curative resection

In terms of RFS, univariable analysis revealed that age (≥65 years; *P* = .0012), sex (male; *P* = .0014), tumor distance from the anal verge (≤5 cm; *P* = .0013), tumor differentiation (moderate or poor; *P* < .0001), lymphatic invasion (present; *P* < .0001), venous invasion (present; *P* < .0001), TNM stage (stage II or III; *P* < .0001), circumferential resection margin (positive; *P* < .0001), and adjuvant chemotherapy (present; *P* < .0001) were significantly associated with recurrence. Multivariable analyses using Cox proportional hazards regression models revealed that older age (HR 1.47, 95% CI 1.19‐1.80; *P* = .0003), male (HR 1.45, 95% CI 1.16‐1.83; *P* = .0014), tumor distance from the anal verge ≤ 5 cm (HR 1.36, 95% CI 1.11‐1.66; *P* = .0026), presence of lymphatic invasion (HR 1.69, 95% CI 1.36‐2.09; *P* < .0001), presence of venous invasion (HR 1.34, 95% CI 1.07‐1.69; *P* = .0127), retrieved LNs < 12 (HR 1.66, 95% CI 1.13‐2.45; *P* = .0096), advanced TNM stage (stage II vs stage I: HR 2.11, 95% CI 1.50‐2.99; *P* < .0001, and stage III vs stage I: HR 3.91, 95% CI 2.81‐5.45; *P* < .0001), and positive circumferential resection margin (HR 2.01, 95% CI 1.34‐3.00; *P* = .0007) were independent predictors of recurrence (Supplementary Table). After adjusting for these clinical factors, 5‐year RFS was worse in the normalized group (HR 1.34, 95% CI 1.04‐1.71; *P* = .0216) compared to the normal group, but did not significantly differ compared to the elevated group (HR 0.76, 95% CI 0.48‐1.22: *P* = .2521) (Supplementary Table).

In terms of overall survival, univariable analysis revealed that age (≥65 years; *P* < .0001), operation year (2000‐2007; *P* = .0006), sex (male; *P* = .0021), tumor distance from the anal verge ( ≤5; *P* = .0284), tumor differentiation (moderate or poor; *P* < .0001), lymphatic invasion (present; *P* < .0001), venous invasion (present; *P* = .0001), TNM stage (stage II or III; *P* < .0001), and adjuvant chemotherapy (present; *P* = .0074) were significantly associated with worse OS. Multivariable analyses using Cox proportional hazards regression models revealed that older age (HR 1.95, 95% CI 1.50‐2.54; *P* < .0001), early phase of operation year (2000‐2007 vs 2008‐2015: HR 1.67, 95% CI 1.21‐2.29; *P* = .0017), male (HR 1.57, 95% CI 1.16‐2.13; *P* = .0033), moderate or poor differentiation (moderate vs well: HR 1.32, 95% CI 1.00‐1.74; *P* = .0479, and poor vs well: HR 1.71, 95% CI 1.06‐2.77; *P* = .0276), presence of lymphatic invasion (HR 1.59, 95% CI 1.20‐2.09; *P* = .0010), advanced tumor stage (stage II vs stage I: HR 1.78, 95% CI 1.14‐2.80; *P* = .0118, and stage III vs stage I: HR 3.11, 95% CI 2.04‐4.74; *P* < .0001), and positive circumferential resection margin (HR 1.79, 95% CI 1.03‐3.12; *P* = .0400) were all independent predictors of worse OS (Table 2). After adjusting for these clinical factors, the normalized group had worse OS (HR 1.45, 95% CI 1.06‐2.00; *P* = .0211) compared to the normal group, but better OS compared to the elevated group (HR 0.55, 95% CI 0.32‐0.95: *P* = .0305) (Table 2).

### 3.6 Recurrence patterns by group

During the study period, 315 (21%) patients developed recurrence. The recurrence rates were 6% (35/564) for stage I patients, 19% (58/313) for stage II patients, and 36% (222/613) for stage III patients.

According to CEA levels, recurrence rates were 18% (222/1208) for the normal group, 33% (77/235) for the normalized group, and 34% (16/47) for the elevated group (Table 3), suggesting that patients with elevated preoperative CEA (either with normalized or elevated postoperative CEA) are more prone to recurrence (*P* < .0001).

Overall local recurrence rate was 5.4% (80/1490). According to CEA levels, 5% (58/1208) in the normal group, 7% (17/235) in the normalized group, and 11% (5/47) in the elevated group developed local recurrence (*P* = .0844).

### 3.7 Propensity score matching

Propensity score matching analysis was conducted to balance the distribution of covariates between the normal preoperative CEA group and elevated preoperative CEA group (which includes the normalized and elevated subgroups). After propensity score matching, 280 matched pairs of patients were selected and patient distributions were balanced between the two groups. In the cohort of matched patients (n = 560), the elevated preoperative CEA group had worse OS than the normal preoperative CEA group (*P* = .0024). Specifically, the 5‐year OS for normal and elevated preoperative CEA groups was 86.7% and 77.2%, respectively.

## 4 DISCUSSION

We demonstrated that in stage I‐III rectal cancer patients who underwent no preoperative therapy, those with preoperatively elevated and postoperatively normalized CEA following resection of rectal cancer had worse prognoses, both in terms of RFS and OS, compared to those with normal preoperative CEA. These findings are quite different from a previous report of a large‐scale study on colon cancer,^9^ which concluded that preoperatively elevated and postoperatively normalized CEA levels is not an indicator of poor prognosis. In contrast, we found that “preoperatively elevated and postoperatively normalized CEA levels” may be a specific indicator of poor prognosis in rectal cancer patients. Thus, the prognostic significance of CEA levels following curative resection of rectal cancer differs from that of colon cancer. On the other hand, our finding that the prognoses of patients with preoperatively elevated and postoperatively normalized CEA were better than those of patients with continuously elevated perioperative CEA is consistent with a previous report.^7^ Moreover, those with elevated postoperative CEA had the highest and earliest peak in hazard function, with a significantly shorter median time to recurrence, followed by those with normalized postoperative CEA, and those with normal preoperative CEA, in this order. Meanwhile, recurrence sites showed no significant differences among the three groups. Taken together, our results suggest that patients with elevated preoperative CEA are at high risk of developing early recurrence, both distant and local, after resection of rectal cancer, regardless of whether levels normalize postoperatively or not.

Controversies exist regarding the survival benefits of intensive follow‐up after curative resection of colorectal cancer.^20^, ^21^, ^22^, ^23^ In rectal cancer alone, several studies have shown that intensive monitoring is beneficial in terms of detecting treatable residual disease such as local recurrence.^22^, ^24^, ^25^ The results of the present study suggest that patients with rectal cancer showing elevated preoperative CEA may require different surveillance from patients with normal preoperative CEA. Intensive follow‐up might allow for detection of treatable residual disease, thereby improving patient outcomes.

In the present study, stage‐specific RFS and OS rates in patients with stage III rectal cancer, but not stage I or II rectal cancer, differed significantly by group, similar to the previous report.^9^ One possible reason for this is the inclusion of patients with falsely elevated CEA in stage I/II, due to the reference range of CEA ≤ 5 ng/ml used in the present study. Indeed, among patients with preoperatively elevated CEA (either with postoperatively normalized or elevated CEA; n =282), median levels of preoperative CEA were 7.4 ng/ml and 9.7 ng/ml in stage I (n = 39) and II (n = 72) patients, respectively, compared to 12.2 ng/ml in stage III (n = 173) patients (*P* = .0037) (data not shown). Thus, stage I/II patients had lower levels of preoperative CEA compared to stage III patients. We analyzed the outcomes using a cutoff CEA level of 10 ng/ml, but results were similar (Figure S2).

The concept of evaluating both preoperative and postoperative CEA levels, which we adopted in this study, might be beneficial in other situations. Recently, several studies have shown that CEA after preoperative chemoradiotherapy is associated with histologic response and prognosis in patients with rectal cancer.^26^, ^27^, ^28^ Those studies suggested that, when combined, pre‐ and post‐chemoradiotherapy CEA could be useful as prognostic factors for disease‐free survival in patients with rectal cancer who undergo treatment with neoadjuvant chemoradiotherapy and curative resection.^26^, ^27^, ^28^ Elevated CEA before preoperative therapy, as well as normalized CEA after preoperative therapy, may also have a specific prognostic impact.

We also analyzed the prognostic significance of serum CA19‐9 levels. Among the 1490 patients of this study cohort, 1472 had information available on preoperative and early postoperative CA19‐9 levels. Similar to the results for CEA, the normalized group had worse OS (n = 103; 5‐year OS 72.7%) compared to the normal group (n = 1350; 5‐year OS 90.7%) and better OS compared to the elevated group (n = 75; 5‐year OS 59.9%) (*P* < .0001) (data not shown), suggesting that normalized CA19‐9 after surgery may also be a specific prognostic indicator.

Our study has some limitations. First, although our large‐scale study cohort was comprised of prospectively collected series of consecutive patients, the study was retrospective in nature and thus may have had selection bias. Second, this study may not directly reflect clinical practice in many Western countries, where neoadjuvant therapy is the current treatment standard. As described above, several studies in Western countries have investigated CEA changes before and after neoadjuvant therapy.^26^, ^27^, ^28^ However, without the knowledge of perioperative CEA changes in rectal cancer in patients who have not undergone preoperative therapy, the results of these studies cannot be fully understood. In this regard, the present study offers important insights for research studies in Japan as well as Western countries, since we examined baseline changes in perioperative CEA in rectal cancer patients without the influence of preoperative therapy. Third, confounders of CEA, such as smoking, liver disease, and diabetes, were not included in our data for analysis, although they might have resulted in false‐positive CEA elevation. Fourth, due to the observational retrospective design, the timing for early postoperative CEA measurement was not consistent across patients. The half‐life of CEA is 3‐5 days, and serum CEA levels decrease to normal levels from 2 weeks to 1 month. Previous studies also showed considerable variation in the timing of early postoperative CEA measurements (7 days to 1 year after surgery).^6^, ^7^, ^29^, ^30^, ^31^ In this study, we focused on “early” postoperative CEA and used measurements obtained within 3 months. This period, however, might not have been long enough for recovery from postoperative complications to occur prior to initiation of adjuvant chemotherapy. Fifth, postoperative CEA measurements were not available in 126 patients, who were subsequently excluded from the analysis, and for this reason, generalizing our findings might not be feasible. Nonetheless, our findings regarding perioperative changes in CEA in rectal cancer patients of the largest cohort to date warrant further consideration.

## 5 CONCLUSION

Patients with elevated preoperative and normalized postoperative CEA following resection of rectal cancer had worse prognoses compared to those with normal preoperative CEA. Unlike previous reports on colon cancer, perioperative changes in CEA might also have a certain prognostic impact in addition to those of preoperative and postoperative CEA. Moreover, patients with elevated preoperative CEA might require intensive follow‐up, especially in earlier periods, regardless of whether levels normalize postoperatively or not, in order to ensure early detection of recurrence after resection.

## CONFLICT OF INTEREST

None declared.

## AUTHOR CONTRIBUTIONS

YN designed the study, collected the data, analyzed and interpreted the data, and prepared the manuscript. DS participated in the design and coordination of the study, analyzed and interpreted the data, and was responsible for writing the manuscript. TT, YT, JI, YA, RS, KM, ST, and YK collected the data, performed the treatments, interpreted the data, and edited the manuscript. All authors read and approved the final manuscript.

## Data Availability

The data that support the findings of this study are available from the corresponding author upon reasonable request.
