# Supplementary figures and images for: Prognostic impact of preoperatively elevated and postoperatively normalized carcinoembryonic antigen levels following curative resection of stage I‐III rectal cancer
Source: Cancer Med. 2019 Dec 4;9(2):653–62. doi: 10.1002/cam4.2758 (PMC6970051; doi:10.1002/cam4.2758)

## Slide 1
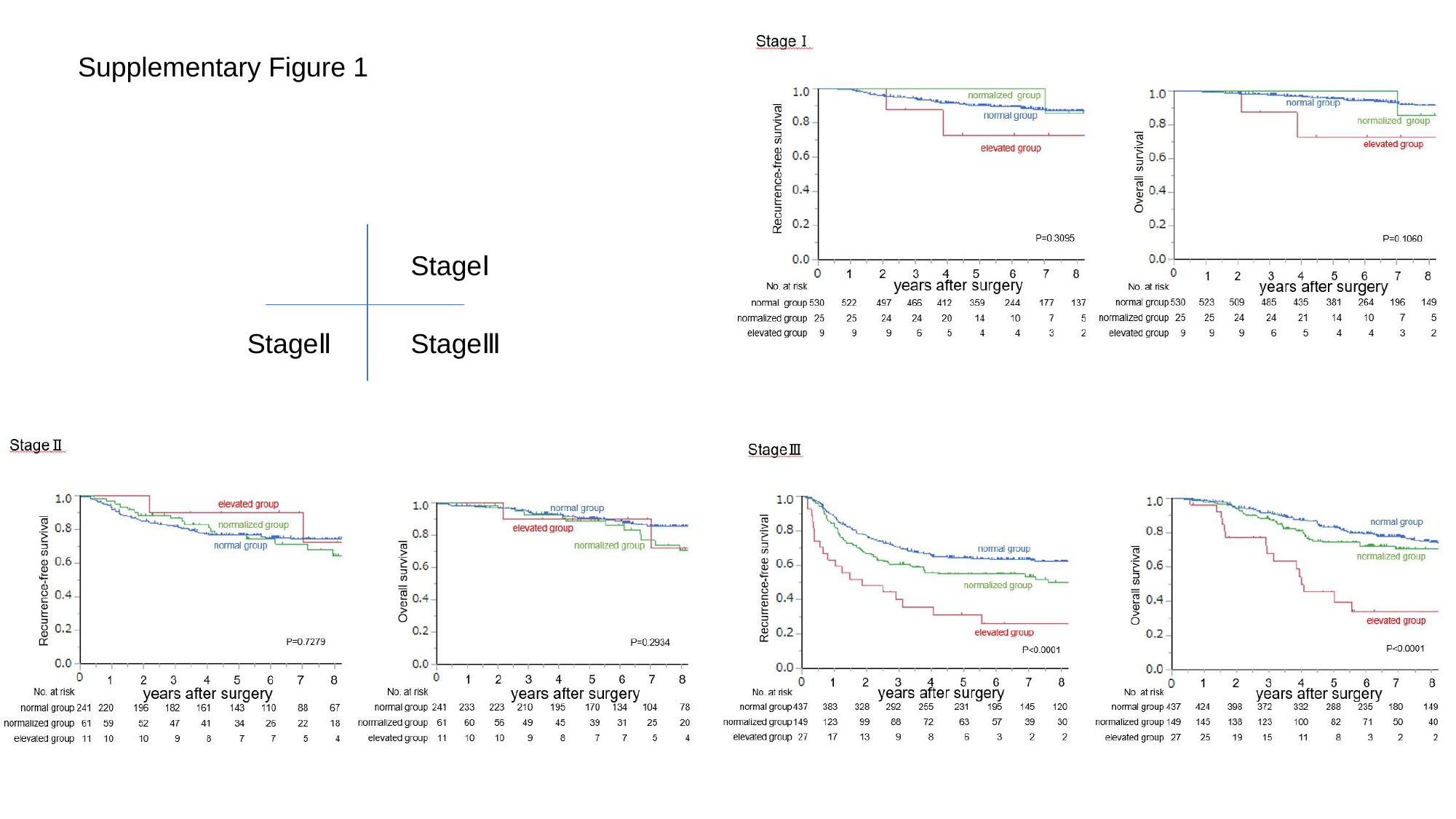

Supplementary Figure 1
StageⅠ
StageⅡ
StageⅢ

Supplement: Supplementary file 1 [file CAM4-9-653-s001.pptx]
