# Supplementary material for: Prognostic impact of preoperatively elevated and postoperatively normalized carcinoembryonic antigen levels following curative resection of stage I‐III rectal cancer
Source: Cancer Med. 2019 Dec 4;9(2):653–62. doi: 10.1002/cam4.2758 (PMC6970051; doi:10.1002/cam4.2758)
Supplement: Supplementary file 2 [file CAM4-9-653-s002.pptx]

## Slide 1
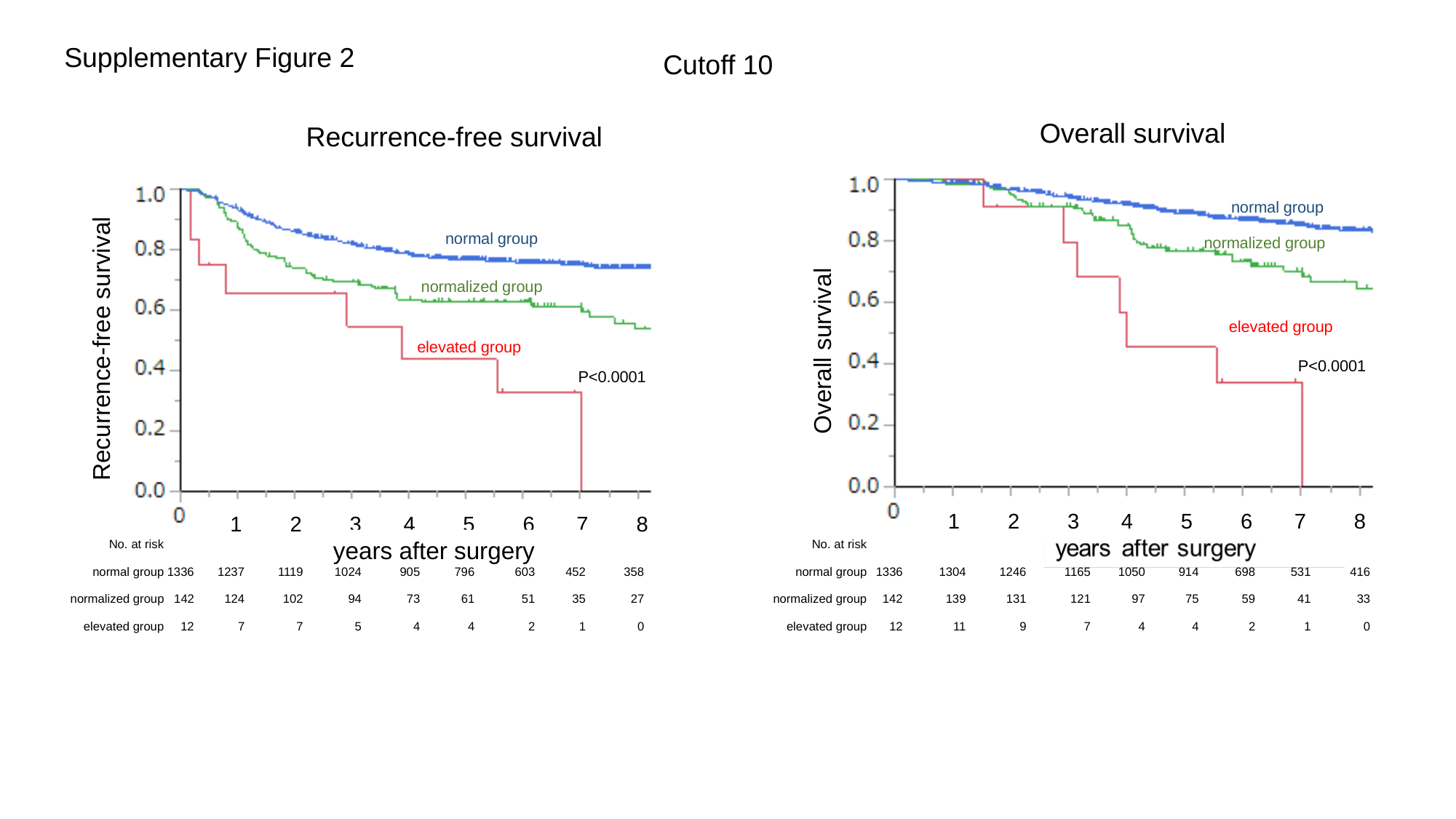

Supplementary Figure 2
Cutoff 10
Overall survival
Recurrence-free survival
normal group
normal group
normalized group
normalized group
elevated group
Recurrence-free survival
Overall survival
elevated group
P<0.0001
P<0.0001
 1 2 3 4 5 6 7 8
 1 2 3 4 5 6 7 8
years after surgery
| No. at risk | | | | | | | | | |
| --- | --- | --- | --- | --- | --- | --- | --- | --- | --- |
| normal group | 1336 | 1304 | 1246 | 1165 | 1050 | 914 | 698 | 531 | 416 |
| normalized group | 142 | 139 | 131 | 121 | 97 | 75 | 59 | 41 | 33 |
| elevated group | 12 | 11 | 9 | 7 | 4 | 4 | 2 | 1 | 0 |
| No. at risk | | | | | | | | | |
| --- | --- | --- | --- | --- | --- | --- | --- | --- | --- |
| normal group | 1336 | 1237 | 1119 | 1024 | 905 | 796 | 603 | 452 | 358 |
| normalized group | 142 | 124 | 102 | 94 | 73 | 61 | 51 | 35 | 27 |
| elevated group | 12 | 7 | 7 | 5 | 4 | 4 | 2 | 1 | 0 |
