# Supplementary material for: Prognostic impact of preoperatively elevated and postoperatively normalized carcinoembryonic antigen levels following curative resection of stage I‐III rectal cancer
Source: Cancer Med. 2019 Dec 4;9(2):653–62. doi: 10.1002/cam4.2758 (PMC6970051; doi:10.1002/cam4.2758)
Supplement: Supplementary file 3 [file CAM4-9-653-s003.docx]

Supplementary Table. Univariable and multivariable analysis of recurrence-free survival

|  | No. of patients | 5-year RFS | 95% CI | *P* | HR | 95% CI | P |
| --- | --- | --- | --- | --- | --- | --- | --- |
| Age, years |  |  |  |  |  |  |  |
| <65 | 906 | 77.4 | 74.5-80.0 | 0.0012 | Reference |  |  |
| ≥65 | 584 | 71.5 | 67.5-75.2 |  | 1.47 | 1.19-1.80 | 0.0003 |
| Operation year |  |  |  |  |  |  |  |
| 2000-2007 | 816 | 74.7 | 71.6-77.6 | 0.6565 | 0.961 | 0.770-1.20 | 0.7216 |
| 2008-2015 | 674 | 75.9 | 72.3-79.1 |  | Reference |  |  |
| Sex |  |  |  |  |  |  |  |
| male | 1001 | 72.5 | 69.5-75.2 | 0.0014 | 1.45 | 1.16-1.83 | 0.0014 |
| female | 489 | 80.5 | 76.7-83.9 |  | Reference |  |  |
| Tumor distance from the anal verge, cm | | |  |  |  |  |  |
| ≤5 | 670 | 70.1 | 66.4-73.5 | 0.0013 | 1.36 | 1.11-1.66 | 0.0026 |
| >5 | 820 | 79.3 | 76.3-82.0 |  | Reference |  |  |
| Tumor differentiation |  |  |  |  |  |  |  |
| well | 803 | 80.2 | 77.2-82.9 | <0.0001 | Reference |  |  |
| moderate | 615 | 70.6 | 66.8-74.2 |  | 1.16 | 0.934-1.43 | 0.184 |
| poor | 71 | 57.0 | 44.8-68.4 |  | 1.26 | 0.841-1.90 | 0.2605 |
| Lymphatic invasion |  |  |  |  |  |  |  |
| yes | 457 | 61.1 | 56.4-65.5 | <0.0001 | 1.69 | 1.36-2.09 | <0.0001 |
| no | 1028 | 81.5 | 78.9-83.8 |  | Reference |  |  |
| Venous invasion |  |  |  |  |  |  |  |
| yes | 828 | 68.3 | 65.0-71.5 | <0.0001 | 1.34 | 1.07-1.69 | 0.0127 |
| no | 656 | 83.6 | 80.5-86.4 |  | Reference |  |  |
| No. retrieved LNs |  |  |  |  |  |  |  |
| ≥12 | 1380 | 75.0 | 72.6-77.3 | 0.8051 | Reference |  |  |
| <12 | 110 | 76.9 | 67.8-84.0 |  | 1.66 | 1.13-2.45 | 0.0096 |
| TNM stage (UICC 8th) |  |  |  |  |  |  |  |
| I | 564 | 90.1 | 87.2-92.4 | <0.0001 | Reference |  |  |
| II | 313 | 77.0 | 71.9-81.5 |  | 2.11 | 1.50-2.99 | <0.0001 |
| III | 613 | 60.5 | 56.5-64.4 |  | 3.91 | 2.81-5.45 | <0.0001 |
| Circumferential resection margin | | |  |  |  |  |  |
| Negative | 1438 | 76.1 | 73.8-78.3 | <0.0001 | Reference |  |  |
| Positive | 52 | 48.1 | 34.6-61.9 |  | 2.01 | 1.34-3.00 | 0.0007 |
| Adjuvant chemotherapy |  |  |  |  |  |  |  |
| yes | 367 | 63.1 | 57.9-68.0 | <0.0001 | 0.789 | 0.609-1.01 | 0.0736 |
| no | 1123 | 79.1 | 76.5-81.5 |  | Reference |  |  |
| CEA group |  |  |  |  |  |  |  |
| normal | 1208 | 78.0 | 75.5-80.3 | <0.0001 | Reference |  |  |
| normalized | 235 | 65.1 | 58.6-71.1 |  | 1.34 | 1.04-1.71 | 0.0216 |
| elevated | 47 | 49.6 | 34.9-64.4 |  | 1.76 | 1.14-2.71 | 0.0106 |

RFS: recurrence-free survival, CI: confidence interval, HR: hazard ratio, LN: lymph node, CEA: carcinoembryonic antigen
